# *Arabidopsis* Seedling Lethal 1 Interacting With Plastid-Encoded RNA Polymerase Complex Proteins Is Essential for Chloroplast Development

**DOI:** 10.3389/fpls.2020.602782

**Published:** 2020-12-16

**Authors:** Deyuan Jiang, Renjie Tang, Yafei Shi, Xiangsheng Ke, Yetao Wang, Yufen Che, Sheng Luan, Xin Hou

**Affiliations:** ^1^State Key Laboratory of Hybrid Rice, College of Life Sciences, Wuhan University, Wuhan, China; ^2^Department of Plant and Microbial Biology, University of California, Berkeley, Berkeley, CA, United States

**Keywords:** Arabidopsis, chloroplast, mitochondrial transcription termination factor, nucleoid, plastid-encoded RNA polymerase

## Abstract

Mitochondrial transcription termination factors (mTERFs) are highly conserved proteins in metazoans. Plants have many more mTERF proteins than animals. The functions and the underlying mechanisms of plants’ mTERFs remain largely unknown. In plants, mTERF family proteins are present in both mitochondria and plastids and are involved in gene expression in these organelles through different mechanisms. In this study, we screened *Arabidopsis* mutants with pigment-defective phenotypes and isolated a T-DNA insertion mutant exhibiting seedling-lethal and albino phenotypes [*seedling lethal 1* (*sl1*)]. The *SL1* gene encodes an mTERF protein localized in the chloroplast stroma. The *sl1* mutant showed severe defects in chloroplast development, photosystem assembly, and the accumulation of photosynthetic proteins. Furthermore, the transcript levels of some plastid-encoded proteins were significantly reduced in the mutant, suggesting that SL1/mTERF3 may function in the chloroplast gene expression. Indeed, SL1/mTERF3 interacted with PAP12/PTAC7, PAP5/PTAC12, and PAP7/PTAC14 in the subgroup of DNA/RNA metabolism in the plastid-encoded RNA polymerase (PEP) complex. Taken together, the characterization of the plant chloroplast mTERF protein, SL1/mTERF3, that associated with PEP complex proteins provided new insights into RNA transcription in the chloroplast.

## Introduction

Plant plastids contain their own genomes that evolved through endosymbiosis as a relic of their cyanobacterial origins. Plastid DNA and the interacting proteins, including RNA polymerase, are organized into plastid nucleoids. Proper expression and functioning of plastid-encoded genes are essential for plant growth and development (Pfannschmidt et al., 2015). The plastid genome of mature chloroplasts typically encodes 75–80 proteins among the estimated 3,500–4,000 proteins present in the chloroplast (Kindgren and Strand, 2015), indicating that the vast majority of the chloroplast proteome is encoded in the nucleus, translated into the cytosol, and subsequently imported into the organelle (Pfannschmidt et al., 2015). Studies have revealed high coordination in the expression of chloroplast proteins encoded by the plastid and nuclear genomes, establishing a concept called genome-coupling (Zhao et al., 2020).

Plastid genes are transcribed by two types of RNA polymerases: the single-subunit, nucleus-encoded plastid RNA polymerase (NEP), and the multi-subunit, plastid-encoded RNA polymerase (PEP; Pfalz and Pfannschmidt, 2013). NEP is responsible for the expression of house-keeping genes, such as *RNA polymerase subunits* (*rpo*) genes, as well as several genes that are involved in gene expression and other basic plastid functions. The *rpo* transcripts are then translated into plastid ribosomes and assembled into PEP. PEP drives the mass production of photosynthesis-related gene transcripts that are necessary for generating functional chloroplasts. Plastid genes can be divided into three classes whose transcription depends solely on PEP (Class I), PEP and NEP (Class II), or NEP alone (Class III; Yu et al., 2014).

Plastid-encoded RNA polymerase is the major transcription machine in the plastid. Earlier biochemical analysis indicated that two different forms of the PEP complex exist in higher plants. PEP-B is composed only of the rpo core subunits (RpoA, RpoB, RpoC1, and RpoC2) and is present in both etioplasts and greening chloroplasts. PEP-A is more complicated and acts as the major RNA polymerase in mature chloroplasts (Pfannschmidt and Link, 1994). The rpo core subunits of PEP are present in both insoluble RNA polymerase preparation, called transcriptionally active chromosome (TAC), and soluble RNA polymerase preparation (sRNAP; Pfalz and Pfannschmidt, 2013). The plastid TAC complex (pTAC) contains 43 nucleus-encoded proteins (Pfalz et al., 2006; Yu et al., 2014), 12 of which are tightly associated with the PEP core and thus are named polymerase-associated proteins (PAPs; Steiner et al., 2011; Pfalz and Pfannschmidt, 2013). The PAPs can be divided into different subgroups depending on their potential functions, including those in DNA/RNA metabolism (PAP1/PTAC3, PAP2/PTAC2, PAP3/PTAC10, PAP5/PTAC12/HEMERA, PAP7/PTAC14, PAP12/PTAC7), redox-dependent regulatory processes (PAP6/Fructokinase-Like 1, Fructokinase-Like 2, PAP10/Thioredoxin Z), reactive oxygen species (ROS) scavenging (PAP4/Fe Superoxide Dismutase 3, PAP9/Fe Superoxide Dismutase 2), and two with unknown function (PAP8/PTAC6, PAP11/MurE-like; Kindgren and Strand, 2015). All PAPs are required for the assembly or stability of the PEP complex (Steiner et al., 2011). *Arabidopsis* knockout lines for each related gene show an albino or pale-green phenotype with severely defected chloroplast development and PEP activity, suggesting that each of these components are required for a functional PEP complex (Steiner et al., 2011; Pfalz and Pfannschmidt, 2013; Pfannschmidt et al., 2015).

In addition to PAPs, other nuclear-encoded proteins, such as mitochondrial transcription termination factors (mTERFs), may play a role in plastid RNA transcription. The mTERF family is so named because the founding member of this protein family, the human mTERF1, promotes transcription termination in human mitochondria (Kruse et al., 1989). The mTERF family in animals has a total of four members, namely, mTERF1–4. mTERF1 and mTERF2 are unique to vertebrates, whereas mTERF3 and mTERF4 are also found in insects and worms, representing more ancestral mTERFs in metazoans (Roberti et al., 2009). Plant genomes encode many more mTERFs than those found in animals (Robles et al., 2012b). In *Arabidopsis*, there are 35 mTERF members, among which 11 mTERF proteins are targeted to chloroplasts and 17 are mitochondrial (Babiychuk et al., 2011). Among the 11 mTERFs located in the chloroplast, several members have been shown to be important in chloroplast RNA processing. For example, *Arabidopsis* mTERF4 or its maize ortholog ZmmTERF4 may be involved in the precise processing of plastid transcripts and may work together with GUN1 in plastid retrograde signaling (Babiychuk et al., 2011; Hammani and Barkan, 2014; Sun et al., 2016). mTERF5 is a transcriptional pausing factor that regulates the transcription of psbEFLJ in the chloroplast (Ding et al., 2019; Meteignier et al., 2020). mTERF6 regulates the maturation of tRNA^Ile^ (GAU; Romani et al., 2015) and transcription termination of the PEP core subunit gene *rpoA* polycistron (Zhang et al., 2018). The functions and underlying mechanisms of many other plant mTERF members, however, remain unknown.

Here, we isolated an albino mutant *seedling lethal 1* (*sl1*) and showed that *SL1* encodes a previously function-unknown mTERF family protein (mTERF3) involved in RNA transcription in the chloroplast. Loss of SL1/mTERF3 led to a severe deficiency in the expression of many plastid proteins and the devastation of photosynthesis. The *sl1* mutant displayed a reduced level of expression in multiple genes transcribed by PEP. SL1/mTERF3 is localized in the chloroplast nucleoid and interacts with PEP accessory proteins PAP12/PTAC7, PAP5/PTAC12, and PAP7/PTAC14, showing that SL1/mTERF3 is involved in RNA transcription in the chloroplast.

## Materials and Methods

### Plant Materials and Growth Conditions

We used *Arabidopsis thaliana* ecotype Columbia-0 in this study. The *A. thaliana* was grown in a growth chamber or green room under 16 h of light at 22°C and 8 h of darkness at 20°C. For soil-grown plants, sown seeds were cold-treated for 2 days and then transferred to the indicated growth conditions. For plate-grown plants, surface-sterilized seeds were planted on 1/2 Murashige and Skoog (MS) medium with 1% (w/v) sucrose and 0.8% (w/v) agar, cold-treated for 2 days, and grown under the same conditions as soil-grown plants. For hydroponic culture, we placed 7-day-old seedlings on 1/2 MS liquid medium prepared with sterilized and deionized water and grown under the same conditions. The nutrient solution was aerated and replaced weekly.

We germinated *Nicotiana benthamiana* seeds on the soil and cultivated them at 25°C under a 14 h light/10 h dark photoperiod with 50–60% relative humidity. We used 4‐ to 5-week-old plants for transient expression analysis.

### Chlorophyll Fluorescence Measurements

We performed chlorophyll fluorescence imaging and analysis with the chlorophyll imaging system FluorCam FC 800-C/1010 (PSI) and determined the photosynthetic parameters as described previously (Hou et al., 2015). Before each measurement, plants were dark-adapted for 20 min. Three independent biological replicates were tested.

### RNA Isolation, cDNA Synthesis, Real-Time Quantitative PCR and Droplet Digital PCR

We isolated total RNA from the leaves using an RNeasy Plant Mini Kit (Qiagen). The cDNA was synthesized using a PrimeScript™ RT Reagent Kit with a gDNA Eraser (Takara). We performed real-time quantitative PCR (RT-qPCR) performed with the 7300Plus RT-qPCR system (ABI) using TB Green™ *Premix Ex Taq*™ II (Tli RNaseH Plus; Takara). We performed droplet digital PCR (ddPCR) with the QX200 ddPCR (ddPCR™) System (Bio-Rad) using ddPCR™ EvaGreen Supermix (Bio-Rad). The *actin2* gene was used as an endogenous control. We determined relative expression levels as previously described (Hou et al., 2009). For each genotype, we performed three independent biological replicates.

### Sequence Comparison and Phylogenetic Analysis

All nucleotide and amino acid sequences of related genes were downloaded from the Arabidopsis Information Resource (TAIR; https://www.arabidopsis.org/) and PLAZA.^1^ We generated a neighbor-joining phylogenetic tree and alignment of mTERF genes from different species using the software of MEGA 5.1 with default settings.

### Plant Transformation and Complementation Analysis

The coding region of the *SL1* gene was amplified by RT-PCR from total RNA and cloned in the vector pHB to produce a construct that expressed the SL1 protein with tags of 3 × Flag driven by a 2 × CaMV 35S promoter (Mao et al., 2005). We used *Agrobacterium tumefaciens* strain GV3101. We transferred the constructs into *SL1/sl1* heterozygous mutant plants using the floral dip method and screened the transgenic positive plants with *sl1/sl1* homozygous backgrounds after transformation. We cloned the *GUS* reporter gene driven by the SL1 promoter into the vector pCAMBIA1381 backbone and transferred it into Columbia-0. We analyzed plants in at least four independent transgenic lines.

### Histochemical GUS Staining

To detect GUS expression in *Arabidopsis*, we incubated samples in 0.1 M sodium phosphate buffer (pH 7.0) containing 1 mg/ml X-Gluc (AMRESCO), 0.5 mM K_3_[Fe(CN)_6_], 0.5 mM K_4_[Fe(CN)_6_], 10 mM Na_2_EDTA, and 0.1% (v/v) Triton X-100, overnight at 37°C in the dark. We cleared GUS-stained tissues with 70% ethanol to remove chlorophyll and stored the tissue in 50% glycerol for examination under the microscope. We observed GUS staining under an SZX12 stereomicroscope (Olympus) and photographed the samples with a digital camera (CoolSNAP, RS photometrics). We conducted three biological replicates for each staining and determined at least three samples for each replicate.

### Protein Preparation, Gel Electrophoresis, and Immunoblot Analysis

Preparation of the total protein sample and thylakoid membranes followed a previous report (Fu et al., 2007). We performed blue native polyacrylamide gel electrophoresis (BN-PAGE) and 2D-SDS PAGE as described in Hou et al. (2015). For immunoblotting analysis, we separated equal amounts of protein sample on 10 or 12% SDS PAGE gels and transferred to nitrocellulose membranes. After blocking nonspecific binding with 5% milk, we subsequently incubated the blot with specific primary antibodies generated against the indicated proteins and secondary horseradish peroxidase-conjugated antibodies. Signals were detected using the SuperSignal™ West Pico PLUS Chemiluminescent Substrate (Thermo Scientific) according to the manufacturer’s protocol. The antibodies used in this study included Anti-PsaD, PsaF, PsbO, PsbP, CytF, Cyt38, ClpC, b6, PetC, ATPα, and PC, which were produced by Dr. Luan (University of California, Berkeley); Anti-D1, D2, and LHCII, which were produced by Dr. A. Melis (University of California, Berkeley; Fu et al., 2007; Che et al., 2013; Hou et al., 2015); and Anti-Flag (Sigma-Aldrich, #F3165), Anti-Tubulin (Sigma-Aldrich, #T8203), Anti-GST (PhytoAB, #PHY5013), and Anti-His (ABclonal, #AE003).

### Microscopic Analyses

We cloned full-length DNA fragments encoding SL1-YFP and PEND-CFP fusion proteins into pCAMBIA1300S to generate the pCAMBIA1300S-SL1-YFP and pCAMBIA1300S-PEND-CFP constructs, respectively (Qiao et al., 2011). We introduced these plasmids into *A. tumefaciens* GV3101 and used them to transform *N. benthamiana* leaves transiently through infiltration. We determined yellow fluorescent protein (YFP) and cyan fluorescent protein (CFP) fluorescence using a confocal microscope FluoView FV1000 (Olympus). Double-labeled cells were scanned sequentially to prevent any cross-talk between fluorescence channels. For transmission electron microscopy (TEM) analysis, we prepared leaves from the wild-type (WT) and *sl1* as described in Yi et al. (2010). We performed observations and image recording using a HT7700 Compact-Digital TEM system (Hitachi). We performed three independent biological replicates.

### Yeast Two-Hybrid Assay

We performed the yeast two-hybrid assay according to the Yeast Protocols Handbook (Clontech). Respective combinations (at least four individual transformants) of the GAL4 DNA binding domain (from pGBKT7) and GAL4 activation domain (from pGADT7) with corresponding proteins were co-transformed into yeast strain Y2H Gold (Clontech). These transformants were grown on SD/-Trp-Leu, SD/-Trp-Leu-His, and SD/-Trp-Leu-His-Ade dropout selective culture medium.

### Protein Expression and GST Pull-Down Experiment

We amplified corresponding genes lacking the N-terminal transpeptide regions and constructed them into the expression vectors pDEST15 and pDEST17 (Invitrogen). The expression vectors pDEST15 and pDEST17 were introduced into the *Escherichia coli* Rosetta and BL21 strain, respectively. These proteins were expressed under 25°C conditions, and pull-down experiments were performed with proteins using a GST affinity column with PBS buffer (pH 7.4). The beads were washed with PBS buffer (pH 7.4), 0.5% Triton X-100, and 0.5 mM PMSF. Bound proteins were eluted and subjected to SDS-PAGE followed by Western blot analysis with the antibody against His tag. We performed three independent biological replicates.

## Results

### Identification of the Albino Mutant *Seedling Lethal 1*

To investigate the gene expression and chloroplast development of the plastid, we generated a T-DNA insertional library by randomly transforming *Arabidopsis* plants (ecotype Columbia-0) with the *A. tumefaciens* strain harboring the pCAMBIA1301 construct, and the mutants were screened by a pigment-defective phenotype, such as albino, pale green, and pale-yellow leaves. Finally, 12 seedling-lethal mutants were obtained from ~4,000 transgenic lines. Among the obtained mutant lines, an albino mutant with the seedling lethal phenotype was characterized and named *sl1*.

**Figure 1:**
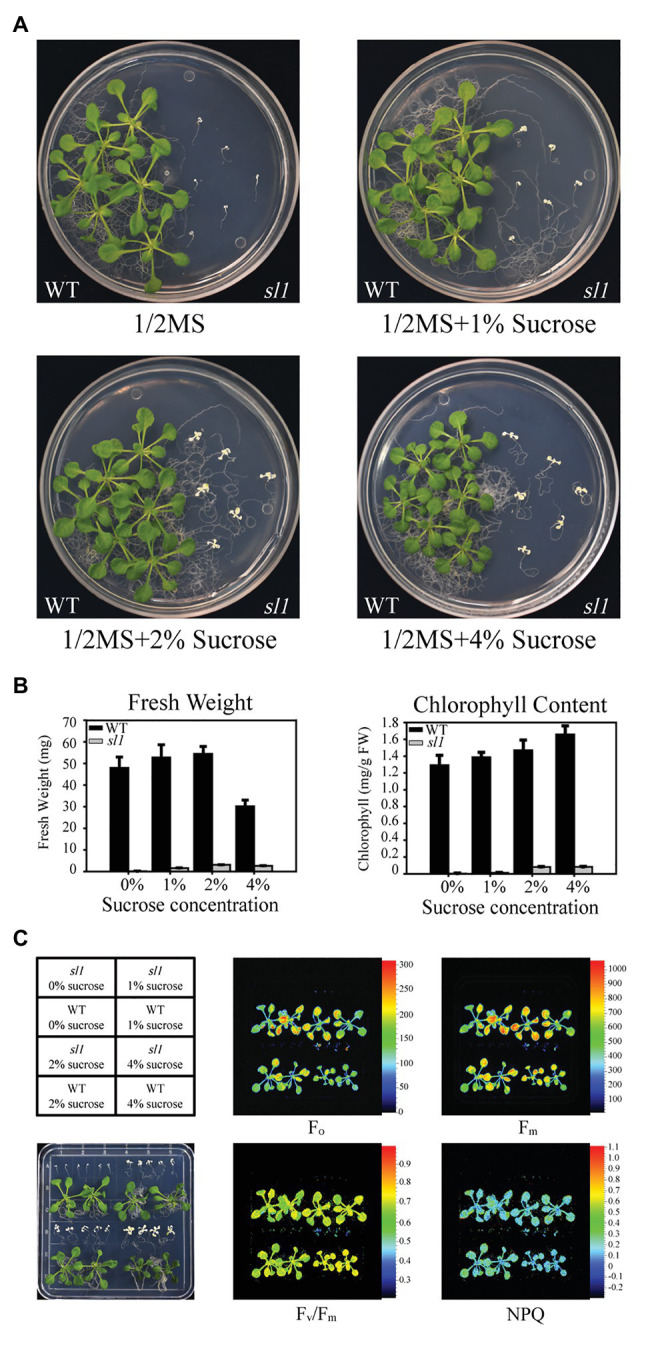
Characterization of the *seedling lethal 1* (*sl1*) mutant. **(A)** Pigment-defective and seedling-lethal phenotypes of the *sl1* mutant. The wild-type (WT) and *sl1* mutant were grown on 1/2 Murashige and Skoog (MS) medium without or with 1, 2, or 4% sucrose (w/v) for 14 days. Experiments were repeated three times, with similar results obtained. **(B)** Fresh weight and chlorophyll content of the WT and *sl1* plants are shown in **(A)**. Three independent biological repetitions with 18 plants were performed for the analysis. **(C)** Chlorophyll fluorescence analysis of the WT and *sl1* plants are indicated in **(A)**. *F_o_*, minimal fluorescence; *F_m_*, maximal fluorescence; *F_v_/F_m_*, maximum efficiency of PSII photochemistry; NPQ, non-photochemical quenching. Three independent biological repetitions with similar results were performed for the analysis.

The *sl1* mutant could germinate on 1/2 MS medium, but it failed to accumulate the pigment, and its growth was arrested at the cotyledon stage (Figure 1A). When supplied with sucrose as a carbon source, the mutant plants grew larger and their leaves produced some green patches (Figure 1A). The fresh weight and chlorophyll content of the *sl1* plants were proportional to the levels of supplemented sucrose (Figure 1B), suggesting that the growth of the *sl1* mutant largely depended on an exogenous carbon source, the absence of which resulted in severe defects in photosynthesis. We measured photosynthetic parameters using chlorophyll fluorescence in seedlings of WT and *sl1* mutant plants grown on 1/2 MS medium supplemented with different concentrations of sucrose. The chlorophyll fluorescence parameters, including *F_o_*: minimal fluorescence, *F_m_*: maximal fluorescence, *F_v_/F_m_*: maximum efficiency of PSII photochemistry, and NPQ: non-photochemical quenching, were substantially lower in the *sl1* mutants than in the WT (Figure 1C), supporting the notion that photosynthesis is impaired in the *sl1* mutant.

### *sl1* Is Mutated in the mTERF3 Gene

**Figure 2:**
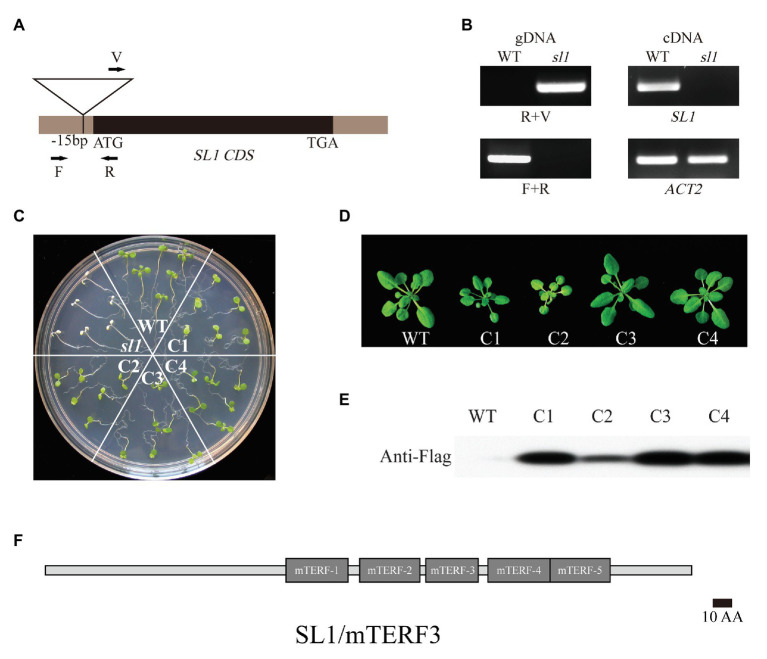
Identification of the knockout line *SL1/mTERF3*. **(A)** Localization of the T-DNA insertion site in *SL1* genomic DNA. Arrows indicate PCR primers. **(B)** Genotyping analysis of *sl1* performed with primers shown in **(A)**. The real-time quantitative (RT-PCR) analysis of *SL1* transcripts in the WT and *sl1*. The *ACTIN2* gene was used as an internal control. **(C)** Molecular complementation of the *sl1* mutant by pHB::SL1-Flag. The 14-day-old WT, *sl1*, and four complemental lines (C1–C4) were grown on 1/2 MS medium with 1% (w/v) sucrose. Three independent biological repetitions were performed. **(D)** WT and four respective complemental lines (C1–C4) plants were grown in the soil for 4 weeks. **(E)** Verification of SL1 expression in complemented lines. Western blot analysis was conducted on total protein extracted from plants as shown in **(D)** using the anti-Flag antibody. **(F)** Schematic representation of SL1/mTERF3 proteins. The numbers and locations of the mitochondrial transcription termination factor (mTERF) domains are shown as gray boxes.

To clone the corresponding *SL1* gene, thermal asymmetric interlaced PCR (TAIL-PCR) was performed to characterize the T-DNA insertion site of the *sl1* mutant (Liu et al., 1995). The sequencing and alignment results showed that T-DNA was inserted into the 5'UTR of locus At2g36000 (Figure 2A and Supplementary Figure S1). The *sl1* mutant represented a homozygous allele of the T-DNA insertion in At2g36000, which showed no detectable full-length mRNA (Figure 2B). Expressing the coding region of At2g36000 fused with the Flag tag rescued the phenotypic defects in the *sl1* mutant, and four independent transgenic plant lines were created (Figures 2C,D). The chlorophyll content and plant size of the different complemented lines were positively correlated to the expression level of the SL1-Flag proteins (Figures 2C–E), confirming that *SL1* was At2g36000.

Sequence alignment and domain architecture analysis using BLAST^2^ and SMART^3^ indicated that SL1 belongs to the mTERFs family and possesses five mTERF domains (MTERFs; Figure 2F). The mTERF family is a large family present in metazoans and plants (Linder et al., 2005), and SL1 was previously named mTERF3 and belongs to the “CHLOROPLAST” cluster (Kleine, 2012).

The homologous features of the *SL1* sequence were analyzed with Dicots PLAZA 4.0.^4^ All 1,133 mTERF-related genes in genomes from different species in the database could be identified with identifier ID IPR003690. The orthologues of *SL1* were widely distributed in Viridiplantae and were found in 53 sequenced genomes of plant species. *SL1* was relatively conserved from *Chlamydomonas reinhardtii* to higher plants, and the phylogenetic tree generated using the *SL1* sequence demonstrated the evolutionary relationships among the various plant species (Supplementary Figure S2).

### SL1 Is Localized in the Chloroplast Stroma

**Figure 3:**
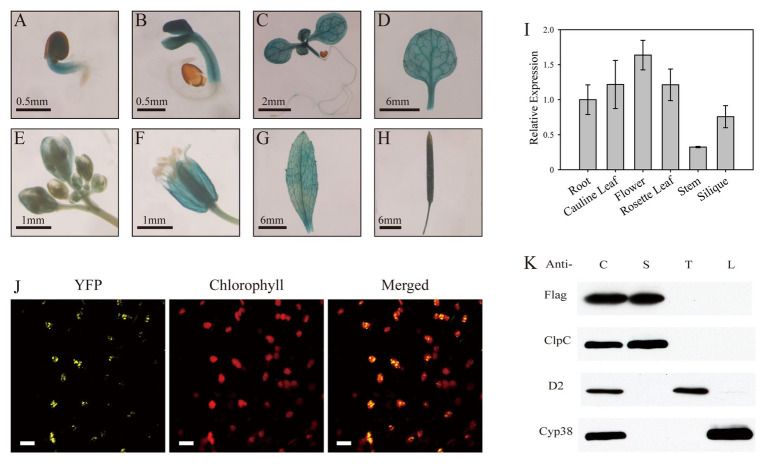
SL1 is expressed in green tissues and localized in the chloroplast stroma. **(A–H)** GUS staining of *SL1_Pro_::GUS* transgenic plants, showing that *SL1* is expressed in the **(A)** germinating seed, **(B)** 3-day-old seedling, **(C)** 7-day-old seedling, **(D)** rosette leaf, **(E)** bud, **(F)** flower, **(G)** cauline leaf, and **(H)** silique. Three independent biological repetitions were performed, and three individual plants were used for each repeat. **(I)** Relative expression of *SL1* in different tissues, including the root, cauline leaf, flower, rosette leaf, stem, and silique, at the heading stage. Experiments were replicated three times, with similar results obtained. **(J)** Confocal microscopic images of the SL1-YFP fusion protein expressed transiently in tobacco epidermal leaf cells. Left to right: yellow fluorescent protein (YFP), chlorophyll, merged image. Bars = 10 μm. **(K)** SL1 is located in the chloroplast stroma. Chloroplasts were purified from SL1-Flag transgenic plants, and the immunoblot analysis was conducted using antibodies against Flag, ClpC (a stroma protein), D2 (a thylakoid membrane protein), and Cyp38 (a thylakoid lumen protein). C, intact chloroplast; S, stroma; T, thylakoid membrane; and L, thylakoid lumen.

To investigate the expression pattern of the *SL1* gene in plants, we generated four independent transgenic plant lines expressing the GUS reporter gene driven by the *SL1* promoter. GUS staining of the transgenic plants showed that in the germinating seeds and seedlings, *SL1* was expressed in the cotyledons and rosette leaves but not in the roots (Figures 3A–C). In adult plants, GUS activity was highly detected in the rosette leaves, flowers, and siliques (Figures 3D–H). In flowers, GUS staining was noted exclusively in the green tissues, such as the sepals, stamens, and carpels, but not in the petals (Figure 3F). The RT-qPCR of the gene expression profile experiments also indicated that *SL1* was expressed in all of the examined tissues and organs, although the expression level was slightly higher in the leaves and flowers (Figure 3I). Above all, *SL1* was expressed in the green aerial tissues.

The mTERF family proteins are mostly localized in the chloroplasts and/or mitochondria (Babiychuk et al., 2011). To determine the subcellular localization of SL1, the coding sequence of SL1 was fused with YFP and transiently expressed in *N. benthamiana* leaves. Confocal microscopy detected that YFP fluorescence was co-localized with chlorophyll autofluorescence (Figure 3J), indicating that the SL1 protein was localized in the chloroplasts.

To further investigate the sub-chloroplast localization of the SL1 protein, intact chloroplasts extracted from SL1-Flag complemented plants [complemented line (C1)] were fractionated to stroma, thylakoid membrane, and thylakoid lumen fractions. ClpC (stroma), D2 (thylakoid membrane), and Cyp38 (thylakoid lumen) were selected as marker proteins. The immunoblot analysis results showed that the Flag-tagged SL1 protein, like ClpC (a well-known stroma protein), was detected only in the stroma fraction (Figure 3K). Taken together, the results implied that the SL1 protein was located in the chloroplast stroma.

### *sl1* Mutant Shows Defects in Chloroplast Development and Photosynthetic Complexes Assembly

**Figure 4:**
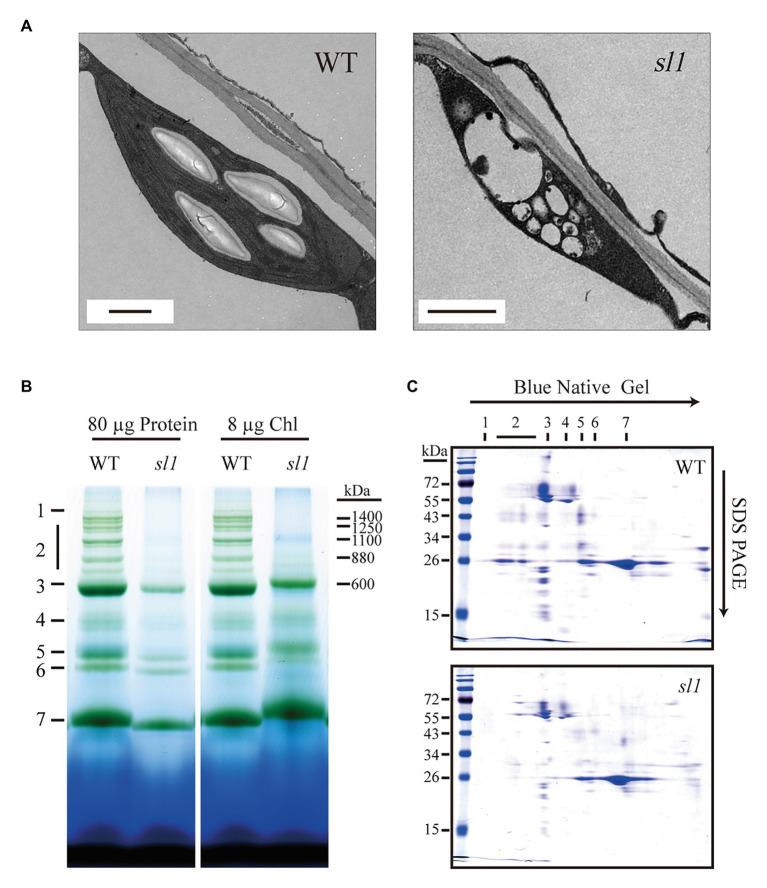
The *sl1* mutant defective in chloroplast development and photosynthetic complexes assembly. **(A)** Electron micrographs of chloroplasts from 10-day-old WT and *sl1* plants grown on 1/2 MS medium with 1% sucrose. Bars = 1 μm. **(B)** Blue native (BN) gel analysis of thylakoid protein complexes in WT and *sl1* (left, 80 μg proteins; right, 8 μg chlorophyll, chl). 1, NDH complex; 2, PSII super complexes; 3, PSI monomer, PSII dimer, and PSII monomer with LHCII trimer; 4, PSI monomer and CF_1_ complex; 5, PSII monomer; 6, LHCII assembly; and 7, LHCII trimer. **(C)** Thylakoid proteins separated by BN gel in **(B)** (equal to 8 μg chlorophyll loading) were further subjected to SDS-PAGE and stained with Coomassie blue. Annotation of the different complexes is indicated in **(B)**.

As the *sl1* mutant is pigment-defective and exhibits suppressed photosynthesis, we examined the morphology and ultrastructure of the chloroplasts in the *sl1* mutants by TEM. Under normal growth conditions, chloroplasts in the WT were lens-shaped and contained well-organized thylakoid membrane systems composed of stroma and grana thylakoids. The chloroplasts in the *sl1* mutants, however, were irregular and much smaller than those in the WT plants and contained a number of vesicles. They did not have thylakoid membranes or any membrane structures and were completely devoid of starch grains (Figure 4A). These results showed that the thylakoid membrane was severely impaired in the *sl1* mutants and that the *SL1* gene was important for thylakoid biogenesis and had a critical role in chloroplast development.

SL1 is essential for chlorophyll production and autotrophic growth, which motivated us to examine the photosynthetic machinery of the mutant. We conducted a BN-PAGE analysis with the WT and *sl1* thylakoid membrane proteins. Because of the heavy damage to chloroplast development in the *sl1* mutant, it was difficult to extract sufficient chloroplast proteins for the BN-PAGE to quantify the loading by total protein amount (Figure 4B, left). To resolve this, we quantified the loading samples by the chlorophyll amount in this analysis (Figure 4B, right), which represented the quantity of chloroplast proteins. The result indicated definite differences in the NDH and PSII super complexes (PSII SCs). The NDH and PSII SCs assembly could hardly be observed in *sl1*. The *sl1* mutant plants were almost totally deficient of PSII SCs and were facilely located in the WT (Figure 4B). Protein identification was completed according to Hou et al. (2015). To further visualize changes in the PSII SCs, when subjecting the BN gel slices to the second electrophoresis dimension by SDS/PAGE, we observed an intense reduction in the abundance of protein subunits associated with PSII SCs (Figure 4C). These results indicated that SL1 had significant functions in the chloroplast photosynthetic complexes assembly.

### *sl1* Is Defective in the Accumulation of Photosynthetic Proteins and Plastid Gene Transcription

**Figure 5:**
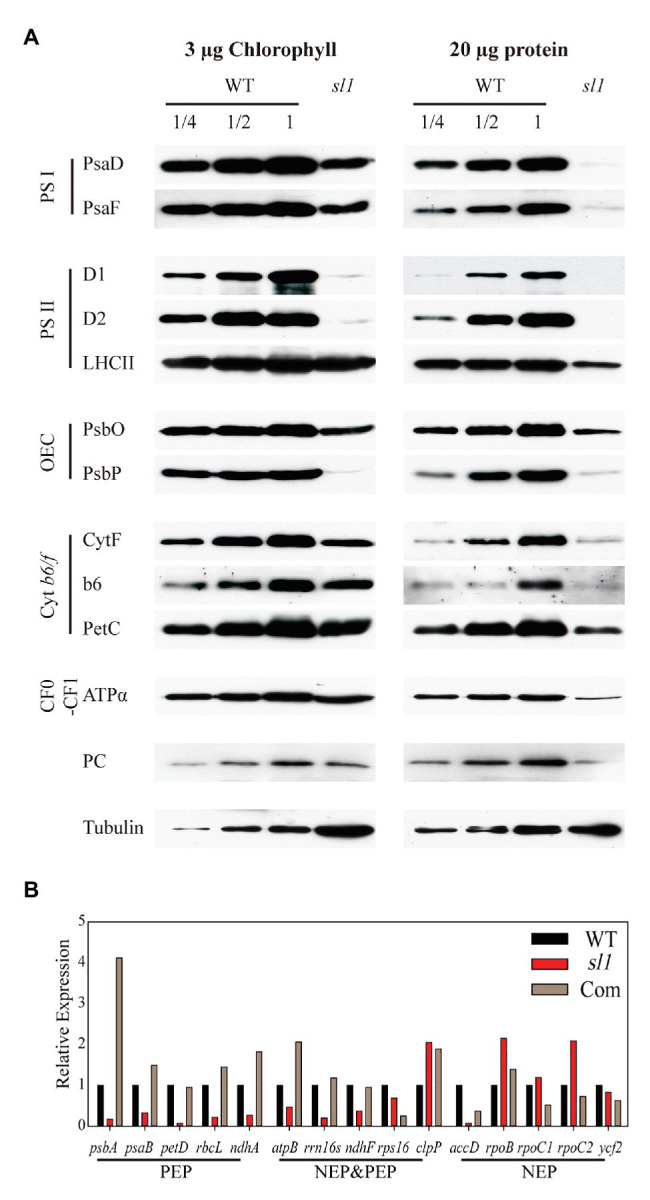
The *sl1* mutant is defective in photosynthetic protein expression and RNA transcription of plastid genes. **(A)** Western blot analysis of photosynthetic proteins in the WT and *sl1* mutant. Protein samples equivalent to 3 μg chlorophyll (left) or 20 μg protein (right) were separated by 10% SDS/PAGE, blotted, and probed with the indicated antibody. Tubulin was used as a loading control. **(B)** Relative expression of plastid genome coding genes [Class I: plastid-encoded RNA polymerase (PEP); Class II: nucleus-encoded plastid RNA polymerase (NEP) and PEP; Class III: NEP] in WT, *sl1*, and complemented line (Com) by absolute quantification of droplet digital PCR (ddPCR).

Lower abundances of photosynthetic proteins may alter photosynthetic complex assembly. It was difficult to compare and analyze the abundance of each individual protein between the WT and *sl1* using the second electrophoresis dimension SDS/PAGE. Thus, to compare the accumulation of chloroplast proteins of the *sl1* mutant with those of the WT, photosynthetic protein amounts were evaluated comparably by loading samples on the same SDS/PAGE gel followed by Western immunoblot analysis. We used antibodies for two members of PSI complexes (PsaD and PsaF), three members of PSII complexes (D1, D2, and LHCII), two members of the oxygen-evolving complex (PsbO and PsbP), three members of the Cyt b6/f-complex (CytF, Cyt b6, and PetC), and ATPα of CF_0_–CF_1_ ATP synthase and plastocyanin. Tubulin was used as a loading control. As shown in Figure 5A, when loading equal amounts of total proteins, the *sl1* mutant almost had no detectable subunits of photosynthetic complexes. When loading equal chlorophyll of the proteins, compared with the WT, the accumulation of photosynthetic complex subunits was reduced in the *sl1* mutant, especially the PSII reaction center protein D1 (PsbA), D2 (PsbD), and oxygen-evolving enhancer protein 2 (PsbP). Therefore, a deficiency in photosynthetic protein accumulation and photosynthetic complex assembly resulted in abnormal chloroplast development in the *sl1* mutant.

Considering that SL1 is an mTERF family protein that is putatively involved in transcriptional control, the relative expression levels of plastid genes in the WT and *sl1* were studied by relative quantification of RT-qPCR. To investigate whether the *SL1* mutation specifically affected transcription by NEP or PEP, the three classes of genes that were transcribed by different RNA polymerases were included, namely, Class I (PEP): *psbA*, *psaB*, *petD*, *rbcL*, and *ndhA*; Class II (NEP and PEP): *atpB*, *rrn16s*, *ndhF*, *rps16*, and *clpP*; and Class III (NEP): *accD*, *rpoB*, *rpoC1*, *rproC2*, and *ycf2* (Supplementary Figure S3). To calculate the accurate expression difference of these genes in *sl1*, the expression levels of plastid genes in the WT, *sl1*, and C1 were analyzed by absolute quantification of ddPCR. As shown in Figure 5B, compared with WT, the expression levels of all Class I genes and most Class II genes were strongly reduced in the *sl1* mutant. Most transcript levels of the Class III genes were not strongly reduced in the *sl1* mutant (Figure 5B). These results indicated that the presence of SL1 was important for plastid gene transcription by PEP.

### SL1 Interacts With PEP Complex Proteins

**Figure 6:**
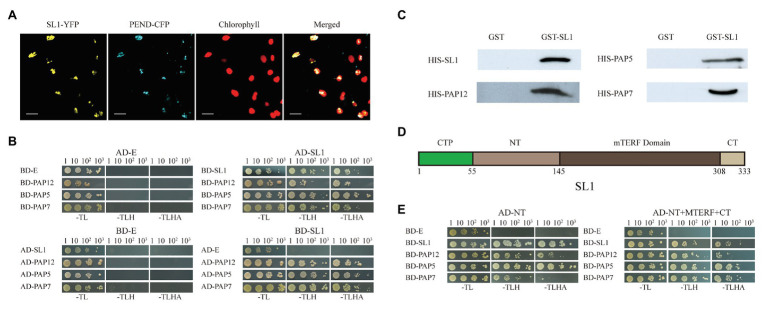
SL1 was localized in the nucleoid and directly interacts with SL1 and the PEP complex proteins PAP12/PTAC7, PAP5/PTAC12, and PAP7/PTAC14. **(A)** Co-localization of SL1 and nucleoid protein PEND. Confocal microscopic images of the SL1-YFP and PEND-CFP fusion proteins co-expressed transiently in tobacco epidermal leaf cells. Left to right: YFP, cyan fluorescent protein (CFP), chlorophyll, merged image. Bars = 10 μm. **(B)** Yeast two-hybrid assay showing that SL1 could interact with SL1, PAP12/PTAC7, PAP5/PTAC12, and PAP7/PTAC14, but no other members of the PEP complex proteins and mTERFs, in the chloroplast (Supplementary Figure S4). AD, the GAL4 activation domain; BD, GAL4 DNA binding domain; E, empty vector. -TL, -TLH, and -TLHA indicate SD/-Trp-Leu, SD/-Trp-Leu-His, and SD/-Trp-Leu-His-Ade dropout medium, respectively. **(C)** GST pull-down assay revealing the interactions of SL1 and its interacting proteins *in vitro*. GST-SL1 and free GST proteins coupled to glutathione resin were incubated with His fusion proteins. Bound proteins were then probed with antibodies against His tag. **(D)** Diagram of the domain structures of SL1. The domains were predicted based on SMART and Pfam. CTP, chloroplast transit peptide; NT, N-terminal of mature SL1; and CT, C-terminal of mature SL1. The numbers indicated amino acids. **(E)** Yeast two-hybrid assay indicating that the NT is essential for protein interactions. The mature protein (right) and the NT (left), but not other parts of SL1 (Supplementary Figure S5), could interact with SL1, PAP12/PTAC7, PAP5/PTAC12, and PAP7/PTAC14. Four independent biological replicates were performed.

Plastid gene transcription occurred at the nucleoid. Similar to bacteria, nucleoids are organized into dense particles in plastids (Pfalz and Pfannschmidt, 2013). While SL1-Flag fusion was localized in the chloroplast stroma, the yellow fluorescence exhibited a distinct punctate distribution pattern in tobacco epidermal cells (Figure 3J), reminiscent of the localization of the chloroplast nucleoid proteins FLN1, FLN2, FSD3, MRL7, and PRDA1, as reported previously (Myouga et al., 2008; Arsova et al., 2010; Qiao et al., 2013). To examine whether SL1 was localized to the chloroplast nucleoid, a co-localization experiment was conducted in which plastid envelope DNA-binding protein (PEND, At3g52170; Sato et al., 1993) was used as a nucleoid protein control. SL1-YFP and PEND-CFP were co-expressed in tobacco epidermal cells and, indeed, the YFP and CFP fluorescent signals overlapped well (Figure 6A), indicating that SL1 was localized to chloroplast nucleoids.

We further explored the mechanism of SL1 by identifying the protein partner for this mTERF protein. As the disruption of SL1 function caused a severe drop in the RNA levels of the plastid genes transcribed by PEP (Figure 5B), and SL1 was located in the nucleoids, we suspected that SL1 may have affected the activity of the PEP complex by interacting with PEP-associated proteins in the nucleoids. We thus tested this possibility by performing a yeast two-hybrid assay using SL1 as bait and all components of the PEP complex (four rpo core proteins and 12 PEP associate proteins, rpoA, AtCg00740; rpoB, AtCg00190; rpoC1, AtCg00180; rpoC2, AtCg00170; PAP2/PTAC2, At1g74850; PAP1/PTAC3, At3g04260; PAP12/PTAC7, At5g24314; PAP3/PTAC10, At3g48500; PAP5/PTAC12, At2g34640; PAP7/PTAC14, At4g20130; PAP6/FLN1, At3g54090; FLN2, At1g69200; PAP10/Trxz, At3g06730; PAP4/FSD2, At5g51100; PAP9/FSD3, At5g23310; PAP8/PTAC6, At1g21600; and PAP11/MurE-Like, At1g63680; Kindgren and Strand, 2015) as prey. These assays showed that SL1 interacted with three PEP-associated proteins, including PAP12/PTAC7, PAP5/PTAC12, and PAP7/PTAC14, but no other PEP complex proteins (Figure 6B and Supplementary Figure S4). We also tested whether SL1 could interact with any of the nine mTERF proteins in the “CHLOROPLAST” cluster (mTERF1/SOLDAT1, At2g03050; mTERF2/EMB221, At2g21710; mTERF3/SL1, At2g36000; mTERF4/BSM/RUG2, At4g02990; mTERF5/MDA1, At4g14605; mTERF6/PDE191, At4g38160; mTERF7, At5g07900; mTERF8/PTAC15, At5g54180; and mTERF9, At5g55580; Kleine, 2012) and found that SL1 interacted with itself but not with other mTERF proteins (Figure 6B and Supplementary Figure S4). These interactions were further confirmed by a GST pull-down assay *in vitro* (Figure 6C).

As SL1 interacted with multiple proteins, it was important to identify the domains responsible for each interaction. The domains were predicted based on SMART and Pfam,^5^ and the SL1 mature protein could be divided into three parts: MTERF in the center position and in the N‐ and C-terminals of mature SL1 (NT and CT). According to these predictions, we constructed a series of SL1 truncations, including chloroplast transit peptide (CTP, amino acid residues 1–164), NT (amino acid residues 165–435), MTERF (amino acid residues 436–924), CT (amino acid residues 925–1,002), and SL1 mature protein (NT+MTERF+CT, amino acid residues 165–1,002, Figure 6D), and examined their interactions with SL1, PAP12/PTAC7, PAP5/PTAC12, and PAP7/PTAC14 using yeast two-hybrid assays. The results showed that the SL1 mature protein (Figure 6E, right) and the NT domain (Figure 6E, left), but not other parts of SL1 (Supplementary Figure S5), interacted with SL1, PAP12/PTAC7, PAP5/PTAC12, and PAP7/PTAC14, indicating that the NT region of SL1 is critical for protein interaction.

## Discussion

In this study, we identified SL1/mTERF3 as an essential protein for chloroplast development and photosynthesis (Figures 4, 5). We provided evidence that SL1/mTERF3 interacts directly with the PEP complex (Figure 6), suggesting that SL1/mTERF3 is an important protein associated with PEP complex proteins that participate in chloroplast gene transcription.

In the PEP complex, all PAPs are essential for chloroplast development and PEP activity in *Arabidopsis* (Steiner et al., 2011; Pfalz and Pfannschmidt, 2013; Pfannschmidt et al., 2015). PAPs can be divided into four groups depending on their potential function: involvement in DNA/RNA metabolism (Group 1), fine-tuning of the redox regulation of chloroplast gene transcription (Group 2), protection of the PEP complex against ROS (Group 3), and unclear function (Group 4; Kindgren and Strand, 2015; Chang et al., 2017). Despite their functional importance, the molecular mechanism of PAPs in plastid transcription is largely unknown (Kindgren and Strand, 2015). The SL1/mTERF3-interacting proteins PAP12/PTAC7, PAP5/PTAC12, and PAP7/PTAC14 belong to the “DNA/RNA metabolism” group of PAPs. PAP5/PTAC12 of *Arabidopsis* has dual localization in the nucleus, as well as in the plastids, and regulates photomorphogenesis (Chen et al., 2010; Galvao et al., 2012; Qiu et al., 2015). Its ortholog in maize, ZmPTAC12, can bind to single-stranded nucleic acids (Pfalz et al., 2015). PAP7/PTAC14 is a SET domain-containing protein that may participate in the transfer of methyl groups on target proteins (Gao et al., 2012). PAP12/PTAC7, PAP5/PTAC12, and PAP7/PTAC14 can interact with one another and form a complex (Gao et al., 2011; Yu et al., 2013), although the functions of PAP12/PTAC7 and PAP7/PTAC14 are not well-defined (Pfannschmidt et al., 2015). PAP2/PTAC2 is required for the proper transcription of psbA (Pfalz et al., 2006). PAP1/PTAC3 belongs to the SAP protein family and is associated with rpo subunits. The *ptac3* mutant exhibits an albino phenotype with reduced PEP-dependent plastid transcription (Yagi et al., 2012). PAP3/PTAC10 can interact with several subunits of the PEP complex, such as PAP12/PTAC7 and PAP7/PTAC14, and the overexpression of PAP3/PTAC10 enhances the expression of PEP-dependent photosynthetic genes (Chang et al., 2017). Our results showed that SL1/mTERF3 directly interacted with PAP12/PTAC7, PAP5/PTAC12, and PAP7/PTAC14 (Figure 6). Accordingly, SL1/mTERF3 may affect plastid gene transcription through these PAPs by directly interacting with them. However, the phenotype of *sl1* mutant has a noteworthy difference to some recent *pap* mutants (Pfalz et al., 2006; Gao et al., 2011; Yu et al., 2013), indicating that the defect of SL1/mTERF3 is not equivalent with PAP defects. This result implied that SL1/mTERF3 also may have other important functions that are independent of the interaction with PAPs.

Mitochondrial transcription termination factor proteins interact with the mitochondrial chromosome and act as a key factor in the regulation of transcription termination in human mitochondria. The mTERF proteins have been identified in metazoans and plants but not in fungi (Roberti et al., 2009; Kleine and Leister, 2015). Their founding member is human mTERF1 (Kruse et al., 1989), which was previously believed to promote transcription termination in human mitochondria. Nevertheless, this assumption was based on *in vitro* studies and was subsequently revised, and mTERF1 is now thought to act as a replication-fork barrier (Shi et al., 2016; Leister and Kleine, 2020). Curiously, it seems that none of those mammalian mTERFs have actual functions in terminating transcription (Kleine and Leister, 2015; Leister and Kleine, 2020). During the course of evolution, plants have developed strategies to adapt to biotic and abiotic stresses, which might have led to the scale expansion and functional diversification of conserved gene families. Four mTERFs are present in mammals, whereas plants have developed many more mTERF proteins than animals (Robles et al., 2012b; Kleine and Leister, 2015; Quesada, 2016). The deficiency of mTERF1, mTERF2, mTERF5, or mTERF9 will inhibit development (Tzafrir et al., 2004; Meskauskiene et al., 2009; Robles et al., 2012a, 2015). mTERF6, mTERF10, and mTERF11 are involved in the response to abiotic stress (Robles et al., 2015, 2018; Xu et al., 2017). Therefore, the specific function of mTERF family members in plants is still largely unknown. Our results showed that the deficiency of SL1/mTERF3 could lead to the complete loss of the photosystem (Figures 4, 5). SL1/mTERF3 is essential for chloroplast development (Figure 4A) and plant growth (Figure 1), indicating that SL1/mTERF3 may be one of the most important mTERF members.

Furthermore, the molecular functions of plant mTERFs are not well-known. Only some mTERF proteins in plants have been reported. Their functions are related to organellar gene expression. mTERF5 and mTERF6 function in organelle gene transcription (Romani et al., 2015; Sun et al., 2016; Zhang et al., 2018; Ding et al., 2019), while mTERF4, mTERF15, and mTERF22 are involved in transcription and RNA splicing (Babiychuk et al., 2011; Hsu et al., 2014; Shevtsov et al., 2018). Similar to mTERF5, SL1/mTERF3 could interact with the PEP complex (Figure 6). mTERF5 interacted with PAP8/PTAC6, controlling RNA transcriptional pausing (Ding et al., 2019). SL1/mTERF3 interacted with PAP12/PTAC7, PAP5/PTAC12, and PAP7/PTAC14 in the PEP complex DNA/RNA metabolism subgroup, and the transcription of Class I genes was significantly affected in the *sl1* mutant (Figure 5B). These results indicated that SL1/mTERF3 has a vital function in chloroplast RNA transcription. Similar to mTERF5/MDA1, mTERF6, and mTERF8/PTAC15, SL1/mTERF3 may have a precise and specific function of the nucleic acid binding ability. The N-terminal part of mature SL1/mTERF3 provided protein-interacting sites with these PAPs (Figure 6), and the MTERF conserved with mTERFs in animals may be involved in RNA transcriptional termination.

The expression of *accD*, which belongs to Class III, was also remarkably reduced in the *sl1* mutant, similar to the Class I genes (Figure 5B). This result implied that SL1/mTERF3 might not only participate in RNA transcription but also has other functions in chloroplast gene expression. For instance, *accD* transcripts have two RNA editing sites in the chloroplast (Chateigner-Boutin and Small, 2007). SL1/mTERF3 may play vital roles in different steps of the transcriptional and post-transcriptional regulation of chloroplast gene expression, such as transcription termination, RNA stabilization, RNA editing, and RNA splicing or even translation. RNA metabolism events, including RNA synthesis, RNA splicing, and RNA editing, may form a unified whole system rather than separate parts. Therefore, besides the PAPs, SL1/mTERF3 may have spatiotemporally direct or indirect interaction with additional key proteins in the spliceosome, editosome, or other complexes and may play a significant role in the comprehensive protein interaction network in plastids.

Although there is some understanding of the PEP core and core-associated components of the complex, the structural and functional mechanism of the PEP complex has not yet been properly explored. In addition to the PEP complex, many other nucleus-encoded proteins, such as mTERFs, are also involved in plastid gene expression and establish an extraordinarily complicated molecular interaction network in the formation and maintenance of the PEP complex. These mTERF members may have different roles in RNA transcription or other processes. Our discovery will add to the understanding of the role of mTERFs in chloroplast RNA metabolism. The *sl1* mutant displayed a seedling-lethal phenotype and had drastically reduced levels of expression of many plastid genes, suggesting that SL1/mTERF3 most likely has a precise and specific function in the regulation of RNA metabolism in plastids. The specific function of SL1/mTERF3, however, remains unknown. We will evaluate the molecular mechanism of SL1/mTERF3 in a future study.

## Data Availability Statement

The original contributions presented in the study are included in the article/Supplementary Material; further inquiries can be directed to the corresponding authors.

## Author Contributions

SL and XH designed research. DJ, RT, YS, XK, YW, YC, and XH performed research. DJ, RT, and XH analyzed data. DJ, SL, and XH wrote the manuscript. All authors contributed to the article and approved the submitted version.

### Conflict of Interest

The authors declare that the research was conducted in the absence of any commercial or financial relationships that could be construed as a potential conflict of interest.

## Footnotes

^1^https://bioinformatics.psb.ugent.be/plaza/

^2^http://www.ncbi.nlm.nih.gov/BLAST/

^3^http://smart.embl-heidelberg.de

^4^https://bioinformatics.psb.ugent.be/plaza/versions/plaza_v4_dicots/

^5^http://pfam.xfam.org/family/PF02536
