# Supplementary material for: Arabidopsis Seedling Lethal 1 Interacting With Plastid-Encoded RNA Polymerase Complex Proteins Is Essential for Chloroplast Development
Source: Front Plant Sci. 2020 Dec 16;11:602782. doi: 10.3389/fpls.2020.602782 (PMC7772139; doi:10.3389/fpls.2020.602782)
Supplement: Supplementary file 1 [file Data_Sheet_1.pdf]

Supplementary Data:

5’ sequence in *sl1*

CAGTACTAAA	ATCCAGATCC	CCCGAATTAA	TTCGGCGTTA	ATTCAGTACA
TTAAAAACGT	CCGCAATGTG	TTATTAAGTT	GTCTAAGCGT	CAATTTGTTT
ACACCACAAT	ATATCCTGAA	TAATTCAACA	AGAAGAATGT	CTCTTGAACA
ATGCAACAAG	AAGCTCTCTC	TTTCCTCTCG	TCCTCTCTCC	CTTCCCTTCA
CCACAATTTT	CCCTCTCTTT	CTCGCCTCCG	CTTCAACAAT	TTTCCCGCAC
TTTCCTTCAA	ACCAAACACT	TCATCATTAT	CTTCATCATT	TCTTCAAATC

Figure S1. Flanking sequences of the transfer DNA (T-DNA) insertion in the *sl1* mutant.

Red, T-DNA insertion sequence; Black, genomic 5’ UTR sequence; Gray, *SL1* CDS sequence.

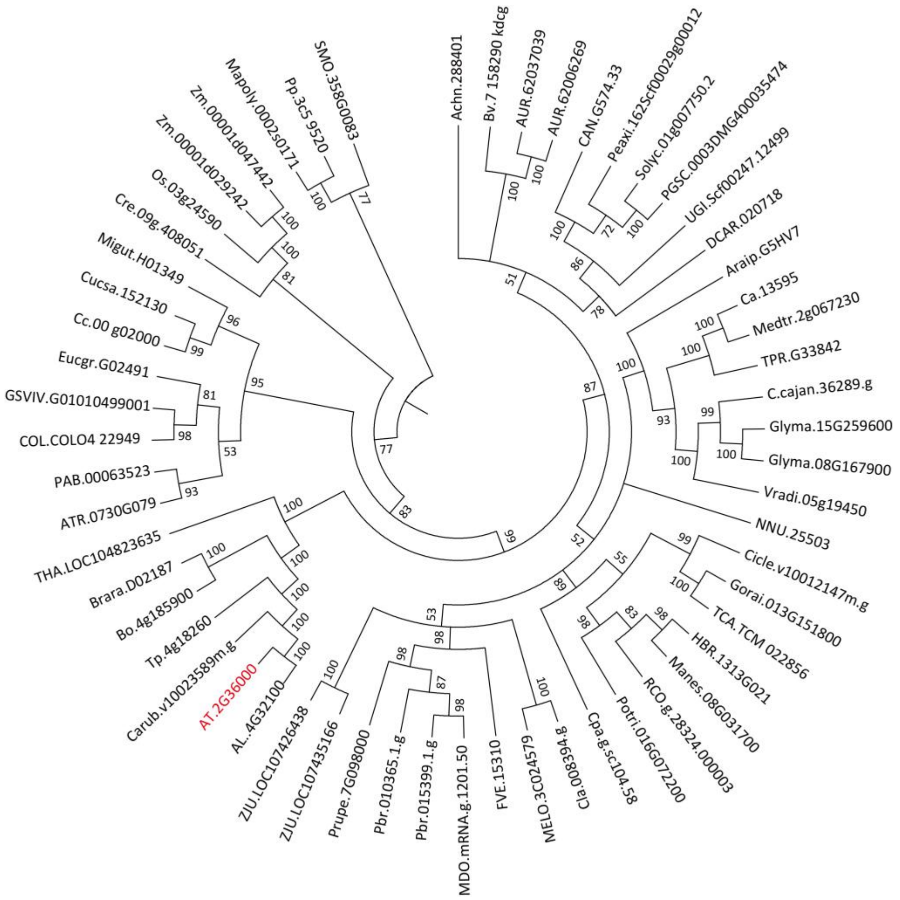

Figure S2. Neighbor-joining phylogenetic tree of *SL1* ortholog genes in plants.

Species abbreviations are Achn, *Actinidia chinensis*; ATR, *Amborella trichopoda*; AL, *Arabidopsis lyrata*; Araip, *Arachis ipaensis*; AT, *Arabidopsis thaliana*; Bv, *Beta vulgaris*; Bo, *Brassica oleracea*; Brara, *Brassica rapa*; C.cajan, *Cajanus cajan*; Carub, *Capsella rubella*; CAN, *Capsicum annuum*; Cpa, *Carica papaya*; AUR, *Chenopodium quinoa*; Cre, *Chlamydomonas reinhardtii*; Ca, *Cicer arietinum*; Cla, *Citrullus lanatus*; Cicle, *Citrus clementina*; Cc, *Coffea canephora*; COL, *Corchorus olitorius*; MELO, *Cucumis melo*; Cucsa, *Cucumis sativus L.*; DCAR, *Daucus carota*; Migut, *Erythranthe guttata*; Eucgr, *Eucalyptus grandis*; FVE, *Fragaria vesca*; Glyma, *Glycine max*; Gorai, *Gossypium raimondii*; HBR, *Hevea brasiliensis*; MDO, *Malus domestica*; Manes, *Manihot esculenta*; Mapoly, *Marchantia polymorpha*; Medtr, *Medicago truncatula*; NNU, *Nelumbo nucifera*; Os, *Oryza sativa ssp. japonica*; Peaxi, *Petunia axillaris*; Pp, *Physcomitrella patens*; PAB, *Picea abies*; Potri, *Populus trichocarpa*; Prupe, *Prunus persica*; Pbr, *Pyrus bretschneideri*; RCO, *Ricinus communis*; Tp, *Schrenkiella parvula*; SMO, *Selaginella moellendorffii*; Solyc, *Solanum lycopersicum*; PGSC, *Solanum tuberosum*; THA, *Tarenaya hassleriana*; TCA, *Theobroma cacao*; TPR, *Trifolium pratense*; UGI, *Utricularia gibba*; Vradi, *Vigna radiata var. radiata*; GSVIV, *Vitis vinifera*; Zm, *Zea*

mays; and ZJU, *Ziziphus jujuba*. Numbers on the nodes refer to bootstrap support values. Integrative orthologs data of *SL1* was from PLAZA.

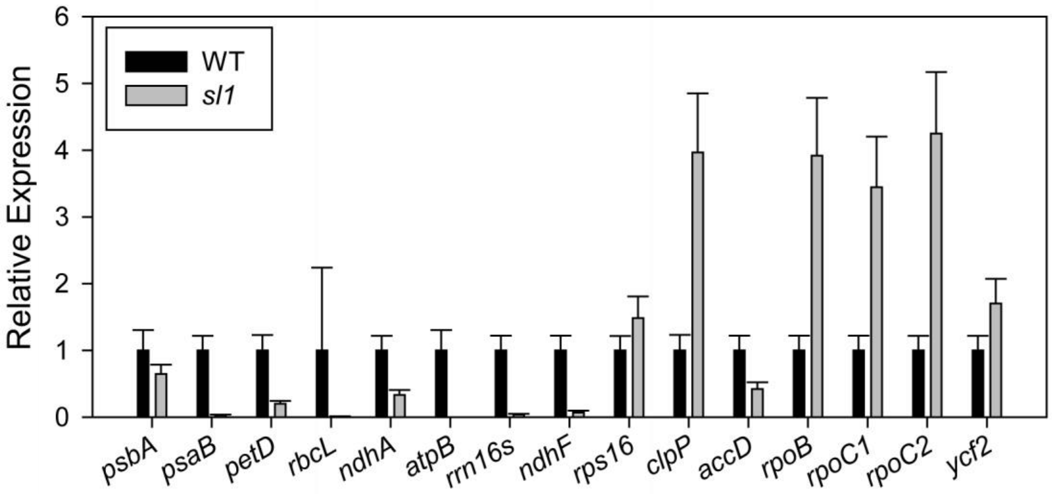

Figure S3. Relative expression of plastid genome coding genes in WT and *sl1* by relative quantification of RT-qPCR. Three independent biological replicates were performed.

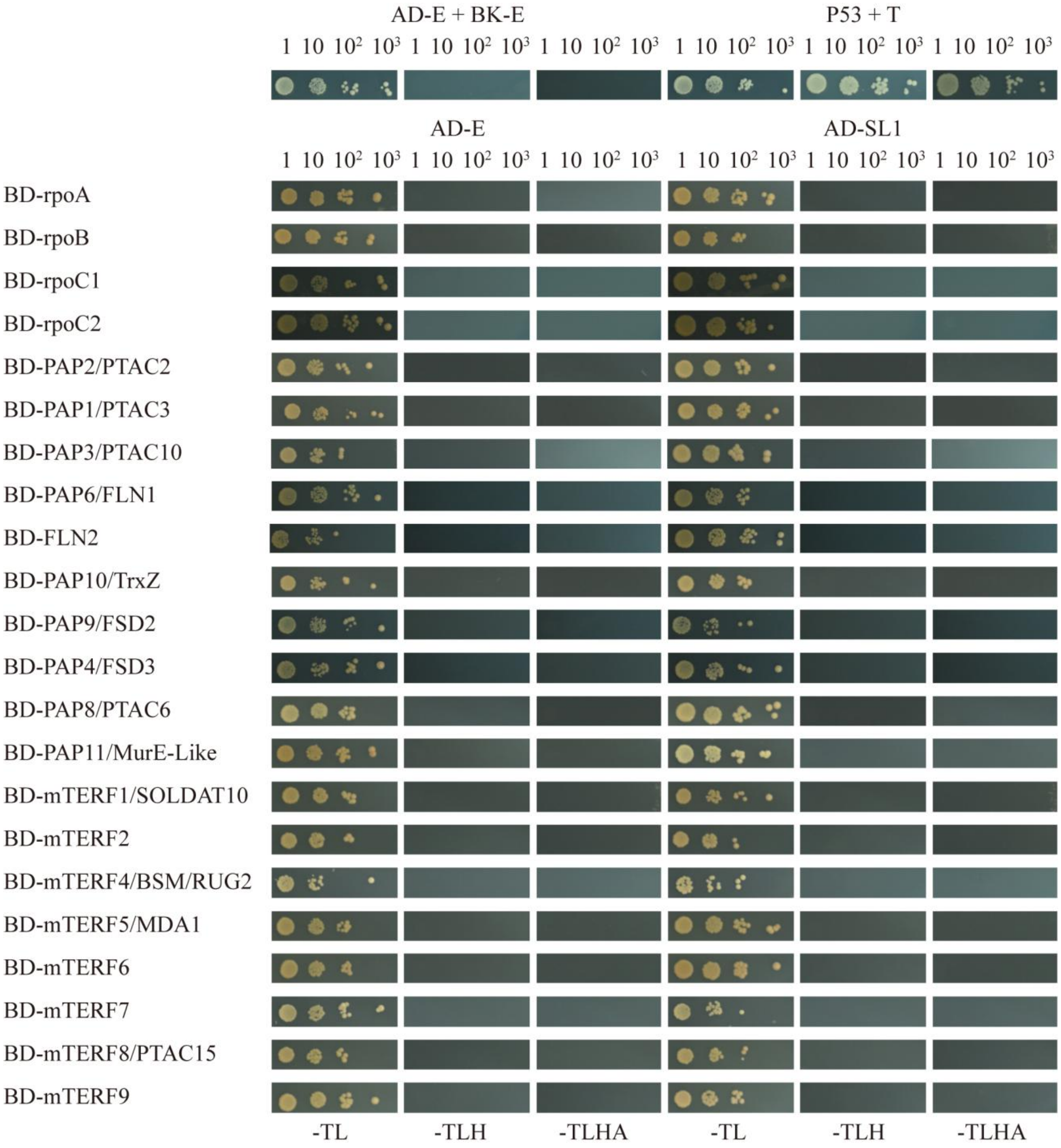

Figure S4. Protein interaction screen of the PEP complex and mTERFs in the chloroplast by yeast two-hybrid assay.

SL1 could interact with SL1, PAP12/PTAC7, PAP5/PTAC12, and PAP7/PTAC14 (**Figure 6B**), but no other PEP complex proteins, or other mTERF family proteins. The AD-E+BK-E was negative control, P53+T was positive control. Four independent biological replicates were performed.

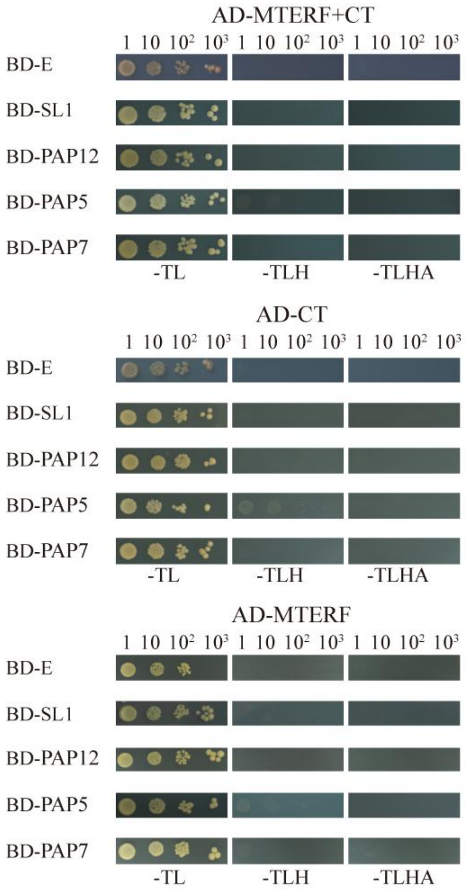

Figure S5. The N-terminal part of the mature SL1 protein is essential for protein interactions.

Yeast two-hybrid assay indicated the N-terminal part of SL1 (**Figure 6E**), but no other parts of SL1, could interact with SL1, PAP12/PTAC7, PAP5/PTAC12, and PAP7/PTAC14. Four independent biological replicates were performed.

Table S1. Primers used in this study.

Names	Primers
SL1-TDNA-F	CCCTTCTCCTTCTCCAAAAATC
SL1-TDNA-R	GAGAGAGCGGTGGAGAGGAGAG
SL1-TDNA-V	AGTACTCGCCGATAGTGGAAACCGACGC
SL1-CDS-F	ATGCAACAAGAAGCTCTCTCTTTC
SL1-CDS-R	AGAATCTTTTTGTACAGTAACAAGGC
SL1-CDS-BP-F	GGGGACAAGTTTGTACAAAAAAGCAGGCTAT GCAACAAGAAGCTCTCTCTTTC
SL1-CDS-BP-R	GGGGACCACTTTGTACAAGAAAGCTGGGTAGA ATCTTTTTGTACAGTAACAAGGC
SL1-qRT-F	GATGGTGGAGATGGGGAGAGATG
SL1-qRT-R	AAAATGTAGGCAGAGGCTGGTTTC
SL1-CDS-KPNI-F	GGGGTACCATGCAACAAGAAGCTCTCTCTTTC
SL1-CDS-SPEI-R	GGACTAGTAGAATCTTTTTGTACAGTAACAAG GC
SL1-Promoter-F	ATATAGTTCATTTGTATCTTAG
SL1-Promoter-R	ATGTGAGATGTCTCTTGAACA
qRT-psbA-F	GAGCAGCAATGAATGCGATA
qRT-psbA-R	CCTATGGGGTCGCTTCTGTA
qRT-psaB-F	GGACCCCACTACTCGTCGTA
qRT-psaB-R	ATTGCTAATTGCCCGAAATG
qRT-petD-F	TCCTTTTGCAACTCCTTTGG
qRT-petD-R	CCGCTGGTACTGAAACCATT
qRT-rbcL-F	GTGTTGGGTTCAAAGCTGGT
qRT-rbcL-R	CATCGGTCCACACAGTTGTC

qRT-ndhA-F	TGCAGGGATACAACAACGTATTG
qRT-ndhA-R	TCGGCTAGAGCTTGAAGAATTCC
qRT-atpB-F	CCGTTTCGTACAAGCAGGAT
qRT-atpB-R	CGGGGTCAGTCAAATCATCT
qRT-clpP-F	GTCGGAGGAGCAATTACCAA
qRT-clpP-R	GTGATGGTTTCGCGAAGTTT
qRT-rrn16S-F	CGGTATCTGGGGAATAAGCA
qRT-rrn16S-R	GATTTGACGGCGGACTTAAA
qRT-ndhF-F	CGGCGGGTATTTTTCTTGTA
qRT-ndhF-R	GGCTAAACCCCGCTTAATGT
qRT-rps16-F	AATCCTTGCAATTGATGTTCGA
qRT-rps16-R	GGATCATAAAAACCCACCTTACTAAGA
qRT-accD-F	TGTGGATTCAATGCGACAAT
qRT-accD-R	TTTTGCGCAGAGTCAATACG
qRT-rpoB-F	AAAAAGCACGGATACGGATG
qRT-rpoB-R	CTTCTTGAATGCCCCGATTA
qRT-rpoC1-F	TCGGATACGAAGATATCAAATGG
qRT-rpoC1-R	TTAGTTATGGGCCTAGCAAAAGA
qRT-rpoC2-F	CCATTTTCCGGACAAGACAGA
qRT-rpoC2-R	CTCTTCCTGGCGGTATCAAGA
qRT-ycf2.1-F	TCCGCAAGGGCCATGAT
qRT-ycf2.1-R	GCTGTCCCGAATTCAAGTTGA
qRT-Actin2-F	GGTAACATTGTGCTCAGTGGTGG
qRT-Actin2-R	CTCGGCCTTGGAGATCCACATC
Y2H-RPOA-BP-F	GGGGACAAGTTTGTACAAAAAAGCAGGCTCTA TGGTTCGAGAGAAAGTCAA

Y2H-RPOA-BP-R	GGGGACCACTTTGTACAAGAAAGCTGGGTATT TTTTTTCTAGAATGTCTAATATCTT
Y2H-RPOB-BP-F	GGGGACAAGTTTGTACAAAAAAGCAGGCTCTA TGCTTGGGGATGAAAAAGAGGGA
Y2H-RPOB-BP-R	GGGGACCACTTTGTACAAGAAAGCTGGGTAAA CTTCCTTCCTATTAATCTGGAAATTCTTCTC
Y2H-RPOC1-BP-F	GGGGACAAGTTTGTACAAAAAAGCAGGCTCTA TGATCGATCGGTATAAACATCAA
Y2H-RPOC1-BP-R	GGGGACCACTTTGTACAAGAAAGCTGGGTAGG TATCATATGAACAGGCTTGAGAA
Y2H-RPOC2-BP-F	GGGGACAAGTTTGTACAAAAAAGCAGGCTCTA TGGCGGAACGGGCCAATC
Y2H-RPOC2-BP-R	GGGGACCACTTTGTACAAGAAAGCTGGGTAAA TCCTAGAAAAGTCAGATTTGGAAATAGAAG
Y2H-PTAC2-BP-F	GGGGACAAGTTTGTACAAAAAAGCAGGCTCTA TGAACCTAGCAATTCCAAATCC
Y2H-PTAC2-BP-R	GGGGACCACTTTGTACAAGAAAGCTGGGTAAG CTGTGCTCCCTGCTAGTT
Y2H-PTAC3-BP-F	GGGGACAAGTTTGTACAAAAAAGCAGGCTCTA TGTCACTCTTGTTCCTCAATCCTCC
Y2H-PTAC3-BP-R	GGGGACCACTTTGTACAAGAAAGCTGGGTAAG AGCTTACTGTTGATGCTGGCT
Y2H-PTAC7-BP-F	GGGGACAAGTTTGTACAAAAAAGCAGGCTCTA TGGCTTCCTTCACCTGTTCT
Y2H-PTAC7-BP-R	GGGGACCACTTTGTACAAGAAAGCTGGGTAAG TGTAGTACTCCAACAAATAGCCAG
Y2H-PTAC10-BP-F	GGGGACAAGTTTGTACAAAAAAGCAGGCTCTA TGCAGATTTGCCAAACCAA
Y2H-PTAC10-BP-R	GGGGACCACTTTGTACAAGAAAGCTGGGTAGT CTGTCAAGACTTGAGTACCGTTA

Y2H-PTAC12-BP-F	GGGGACAAGTTTGTACAAAAAAGCAGGCTCTATGGCGTCAATATCAACCAC
Y2H-PTAC12-BP-R	GGGGACCACTTTGTACAAGAAAGCTGGGTAAGGATCAGTCTCCTCTTCAAAG
Y2H-PTAC14-BP-F	GGGGACAAGTTTGTACAAAAAAGCAGGCTCTATGGCTTCTTCAGTCTCTCTTCAGT
Y2H-PTAC14-BP-R	GGGGACCACTTTGTACAAGAAAGCTGGGTAATAGAGTAACCGTTCTTGATAGATGTCAAGT
Y2H-FLN1-NDEI-F	GGAATTCCATATGATGGCTTCACTTCTTATTTTCCCCC
Y2H-FLN1-XMAI-R	CCCCCCGGGCTACCACATTGATGGAACATAAACTTGTTC
Y2H-FLN2-NDEI-F	GGAATTCCATATGATGGCGTCTCTCTCCTTCACC
Y2H-FLN2-XMAI-R	CCCCCCGGGTCATAAACTACCATCTTCAAACATTGAGCCA
Y2H-TrxZ-BP-F	GGGGACAAGTTTGTACAAAAAAGCAGGCTCTATGGCTCTTGTTCAATCCAGA
Y2H-TrxZ-BP-R	GGGGACCACTTTGTACAAGAAAGCTGGGTACATCTCGTTGTCAATGATATCGT
Y2H-FSD2-BP-F	GGGGACAAGTTTGTACAAAAAAGCAGGCTCTATGATGAATGTTGCAGTGACAG
Y2H-FSD2-BP-R	GGGGACCACTTTGTACAAGAAAGCTGGGTAGTCAACCTCAGATACATCGATGTC
Y2H-FSD3-BP-F	GGGGACAAGTTTGTACAAAAAAGCAGGCTCTATGAGTTCTTGTGTTGTGACGAC
Y2H-FSD3-BP-R	GGGGACCACTTTGTACAAGAAAGCTGGGTAAGCGATTGGGATGTTGG
Y2H-PTAC6-BP-F	GGGGACAAGTTTGTACAAAAAAGCAGGCTCTATGGCGTCTTCCGCCG

Y2H-PTAC6-BP-R	GGGGACCACTTTGTACAAGAAAGCTGGGTAGA GACTTCAGTGTCTCAAATTGGTTC
Y2H-MURE_LIKE-BP-F	GGGGACAAGTTTGTACAAAAAAGCAGGCTCTA TGGCGTTCACCTTTCTCTCT
Y2H-MURE_LIKE-BP-R	GGGGACCACTTTGTACAAGAAAGCTGGGTAAT GACTCTCTGGTAACCGCC
Y2H-PPO1-BP-F	GGGGACAAGTTTGTACAAAAAAGCAGGCTCTA TGGAGTTATCTCTTCTCCGTCC
Y2H-PPO1-BP-R	GGGGACCACTTTGTACAAGAAAGCTGGGTACT TGTAAGCGTACCGTGACATG
Y2H-MTERF1-BP-F	GGGGACAAGTTTGTACAAAAAAGCAGGCTCTA TGATAGCAAGGTGTTCTCTTTTTCCG
Y2H-MTERF1-BP-R	GGGGACCACTTTGTACAAGAAAGCTGGGTATC TTCTTTCAGCAGACCTAAGACG
Y2H-MTERF2-BP-F	GGGGACAAGTTTGTACAAAAAAGCAGGCTCTA TGCTTCTCCACTGCAACGT
Y2H-MTERF2-BP-R	GGGGACCACTTTGTACAAGAAAGCTGGGTATT CTGTCAAATCCTCTTCTTCTTCCG
Y2H-MTERF4/BSM-BP-F	GGGGACAAGTTTGTACAAAAAAGCAGGCTCTA TGAAGATTAGGTTCTGTAATGGCTT
Y2H-MTERF4/BSM-BP-R	GGGGACCACTTTGTACAAGAAAGCTGGGTATG CAAACTCCTCGTCGTCATCATC
Y2H-MTERF5-BP-F	GGGGACAAGTTTGTACAAAAAAGCAGGCTCTA TGCAAAGTCTTAGTCAACTTGGACC
Y2H-MTERF5-BP-R	GGGGACCACTTTGTACAAGAAAGCTGGGTACA ACAAACCACCACTACTTTGTTCAG
Y2H-MTERF6-BP-F	GGGGACAAGTTTGTACAAAAAAGCAGGCTCTA TGGAGGTGACAAATACGAGCAG
Y2H-MTERF6-BP-R	GGGGACCACTTTGTACAAGAAAGCTGGGTAAA TGTGACCTTTGACTAGGGGC

Y2H-MTERF7-BP-F	GGGGACAAGTTTGTACAAAAAAGCAGGCTCTATGGCGGTCGCAATACCTC
Y2H-MTERF7-BP-R	GGGGACCACTTTGTACAAGAAAGCTGGGTAGAGTTTGGTGCATCCTAGATACAGATTC
Y2H-MTERF8-BP-F	GGGGACAAGTTTGTACAAAAAAGCAGGCTCTATGGTGATCTTATCTCTCGTCTCTTGT
Y2H-MTERF8-BP-R	GGGGACCACTTTGTACAAGAAAGCTGGGTATAAACAAATCATTTCGATATTATCAGCCGC
Y2H-MTERF9-BP-F	GGGGACAAGTTTGTACAAAAAAGCAGGCTCTATGGCGGGTTTCTCACTGTACT
Y2H-MTERF9-BP-R	GGGGACCACTTTGTACAAGAAAGCTGGGTATCCTCTCTTGTCATACTTGTTTGCAAACT
PD-SL1-BP-F	GGGGACAAGTTTGTACAAAAAAGCAGGCTCTCTCTCCTCCACCACAACAAC
PD-SL1-BP-R	GGGGACCACTTTGTACAAGAAAGCTGGGTATCAAGAATCTTTTTGTACAGTAACAA
PD-PTAC7-BP-F	GGGGACAAGTTTGTACAAAAAAGCAGGCTCTTGTGGGATTCAGAGAGATGA
PD-PTAC7-BP-R	GGGGACCACTTTGTACAAGAAAGCTGGGTCAAGTGTAGTACTCCAACAAATAGC
PD-PTAC12-BP-F	GGGGACAAGTTTGTACAAAAAAGCAGGCTGCACAGACAGTGGAAAGTCCA
PD-PTAC12-BP-R	GGGGACCACTTTGTACAAGAAAGCTGGGTTAAGGATCAGTCTCCTCTTCAAA
PD-PTAC14-BP-F	GGGGACAAGTTTGTACAAAAAAGCAGGCTCTGCTTCCGAAGAATCTTCC
PD-PTAC14-BP-R	GGGGACCACTTTGTACAAGAAAGCTGGGTCAATAGAGTAACCGTTCTTGATAGA
Y2H-SL1-NT-BP-F	GGGGACAAGTTTGTACAAAAAAGCAGGCTCTCTCTCCTCCACCACAACAACAAC

Y2H-SL1-NT-BP-R	GGGGACCACTTTGTACAAGAAAGCTGGGTATGGGATTGTGTGTGAGGTTAAAGGT
Y2H-SL1-MTERF-BP-F	GGGGACAAGTTTGTACAAAAAAGCAGGCTCTGTCATCACTTTCCTCCTCCGTG
Y2H-SL1-MTERF-BP-R	GGGGACCACTTTGTACAAGAAAGCTGGGTATCTAAAACCAGCTTCATTAGTCTTCAACATCAC
Y2H-SL1-CT-BP-F	GGGGACAAGTTTGTACAAAAAAGCAGGCTCTGATACATTGGAGGTATGTTGT
Y2H-SL1-CT-BP-R	GGGGACCACTTTGTACAAGAAAGCTGGGTAAGAATCTTTTTGTACAGTAACAA
Y2H-SL1-CTP-BP-F	GGGGACAAGTTTGTACAAAAAAGCAGGCTCTATGCAACAAGAAGCTCTCTCTTTCC
Y2H-SL1-CTP-BP-R	GGGGACCACTTTGTACAAGAAAGCTGGGTAAGAGGGTATATCTGGGGATTTGAAG

Table S2. Expression of plastid genome coding genes in WT, *sl1* and complemented line (Com) by absolute quantification of droplet digital PCR (ddPCR).

Gene ID	copies/μL		
	WT	*sl1*	Com
ACTIN	78600	11380	9500
psbA	1912000	47900	951000
psaB	253900	12000	45600
petD	104800	1080	12000
rbcL	1342000	42100	236000
ndhA	38600	1500	8470
atpB	95000	6420	23600
clpP	31500	9350	7180
rrn16s	61500000	1770000	8750000
ndhF	9670	520	1110
rps16	8170	820	250
accD	8760	95	390
rpoB	5780	1800	970
rpoC1	12820	2210	810
rpoC2	13510	4080	1190
ycf2.1	9030	1080	690
